# Causal effects of inflammation on long-term mortality: A mendelian randomization study

**DOI:** 10.18632/aging.206352

**Published:** 2026-02-05

**Authors:** Eliano P. Navarese, Dean J. Kereiakes, Timothy D. Henry, Marc Brouwer, Giuseppe Talanas, Mehriban Isgender, Michael E. Farkouh, Jacek Kubica

**Affiliations:** 1 Department of Life and Health Sciences, Link Campus University, Rome, Italy; 2 SIRIO MEDICINE Research Network, Nicolaus Copernicus University, Bydgoszcz, Poland; 3 The Christ Hospital and Lindner Research Center, Cincinnati, OH 45219, USA; 4 Radboud University Medical Center, Nijmegen, The Netherlands; 5 Clinical Experimental Cardiology, University of Sassari, Sassari, Italy; 6 Department of Cardiology, Republican Clinical Hospital, Azerbaijan Medical University, Baku, Azerbaijan; 7 Department of Family Medicine, Republican Clinical Hospital, Azerbaijan Medical University, Baku, Azerbaijan; 8 Departments of Academic Affairs and Cardiology, Cedars-Sinai Health System, Los Angeles, CA 90048, USA; 9 Department of Cardiology and Internal Medicine, L. Rydygier Collegium Medicum, Nicolaus Copernicus University, Bydgoszcz, Poland

**Keywords:** mendelian randomization, inflammatory biomarkers, mortality, cardiovascular disease

## Abstract

Background: Interleukin-6 (IL6) signaling plays a key role in inflammation and cardiovascular disease, but its causal effect on long-term mortality remains unclear. We aimed to assess whether genetically proxied levels of IL6, soluble IL6 receptor (IL6R), C-reactive protein (CRP), and growth differentiation factor-15 (GDF15) exert causal effects on long-term all-cause mortality, and to examine potential opposing effects of IL6 and IL6R.

Methods: We conducted Mendelian randomization (MR) using genome-wide association study instruments from >750,000 individuals. The primary outcome was all-cause mortality over a median follow-up of 11.7 years. Secondary outcomes included cardiovascular events and selected non-cardiovascular conditions. Multiple sensitivity analyses were applied to evaluate robustness and directionality.

Results: Genetically higher IL6R levels were associated with reduced mortality (odds ratio (OR) per 1-SD increase: 0.95; 95% CI: 0.91–0.98, *p* = 0.007) and lower risk of atrial fibrillation, coronary artery disease, stroke, and lung cancer. Conversely, higher IL6 levels were linked to increased mortality (OR: 1.05; 95% CI: 1.02–1.08, *p* = 0.002). No significant causal effects were observed for CRP or GDF-15. All findings were consistent across sensitivity analyses.

Conclusions: IL6 and IL6R appear to be biologically opposing causal regulators of human survival: IL6 increases, while IL6R reduces mortality through cardiovascular mechanisms. CRP and GDF15 likely reflect disease risk rather than drive it. These results support IL6R antagonism as a potential strategy for cardiovascular disease prevention.

## INTRODUCTION

Chronic low-grade inflammation is currently recognized as a core driver of cardiovascular disease and mortality [1–3]. Additionally, acute inflammatory episodes contribute to mortality risk through distinct but overlapping pathways, including sepsis, cytokine storm syndromes, and acute cardiovascular events [4]. Elevated circulating markers such as interleukin-6 (IL6), C-reactive protein (CRP) and growth differentiation factor-15 (GDF15) track with higher risks of cardiovascular disease, diabetes, cancer and neurodegeneration, yet their causal role remains uncertain because observational data are prone to confounding and reverse causation [5].

The IL6 axis plays a pivotal role in both acute and chronic inflammatory responses, operating through two distinct pathways: classical signalling via a membrane-bound IL6 receptor and trans-signalling mediated by a soluble IL6 receptor (IL6R), which enables IL6 activity in cells lacking the membrane receptor. While acute IL6 elevation drives immediate inflammatory responses essential for pathogen clearance, chronic IL6 activation promotes sustained inflammation contributing to cardiovascular disease development [6]. These parallel routes can exert opposing effects on cardiovascular homeostasis, so therapeutic strategies that inhibit IL6 or enhance IL6R function may have different implications for cardiovascular health and acute disease management [7]. Specifically, IL6R has been involved in the pathogenesis of major cardiovascular conditions including atrial fibrillation, coronary artery disease, and stroke through its modulation of inflammatory cascades in vascular tissues. In comparison, CRP and GDF15 are well-established systemic inflammation markers, reflecting both acute inflammatory states and chronic inflammatory processes, but whether they actively drive cardiovascular pathology or merely mirror it remains a matter of debate [5, 8].

Mendelian randomization (MR) exploits germline genetic variants as unconfounded proxies for modifiable exposures, offering a quasi-experimental route to infer causality across both acute and chronic inflammatory phenotypes [8, 9]. Although individual Mendelian randomization (MR) studies have linked single inflammatory biomarkers to specific diseases, a multi-marker MR assessment spanning multiple inflammatory pathways and focused on cardiovascular disease and all-cause mortality is still lacking.

## METHODS

### Study design and overview

We conducted a comprehensive two-sample MR study following established guidelines and the STROBE-MR reporting standards [9]. Our analysis used publicly available summary statistics from large-scale genome-wide association studies [10, 11]. All analyses focused on individuals of European ancestry to minimize population stratification and ensure validity of genetic instruments.

### Data harmonization and quality control

Prior to analysis, we implemented rigorous data harmonization procedures to ensure consistency across different GWAS datasets. Effect alleles were harmonized across exposure and outcome datasets to ensure consistent direction of effects. We excluded variants with minor allele frequency (MAF) <0.01, imputation quality score <0.8, or missing essential information (effect size, standard error, or sample size).

Palindromic single-nucleotide polymorphisms (SNPs) (A/T or G/C) were carefully handled using allele frequency information to infer strand alignment. When allele frequencies were ambiguous (close to 0.5), palindromic SNPs were excluded to prevent strand misalignment errors. We also excluded variants in the major histocompatibility complex (MHC) region (chromosome 6: 25–35 Mb) due to complex linkage disequilibrium patterns that could violate MR assumptions.

### Selection of genetic instruments

#### Instrument selection criteria and pruning procedures

Genetic instruments were selected based on genome-wide significance for association with the respective biomarker levels. Because relatively few independent genetic variants are robustly associated with circulating inflammatory proteins, we selected instruments using a significance threshold of *P* < 5 × 10^−5^, combined with stringent LD pruning (r² < 0.001) and retention of variants with F-statistics >10 to avoid weak-instrument bias. This selection increased the precision of the causal estimates while preserving validity given our stringent linkage disequilibrium (LD) pruning (r^2^ < 0.001), strong instrument strength, and extensive pleiotropy checks. To ensure independence of instruments, we performed linkage LD pruning using PLINK v1.90 with the following parameters: window size of 10,000 kb, step size of 1,000 variants, and r^2^ threshold of 0.001. LD calculations were based on the 1000 Genomes Project Phase 3 European reference panel. The strength of genetic instruments was assessed using the F-statistic.

We also performed MR-Steiger filtering to ensure that the genetic instruments had stronger associations with the exposure than with the outcome, thereby supporting the assumed causal direction. Additionally, by reverse Mendelian randomization (MR) analysis we tested whether genetic liability to atrial fibrillation (AF), coronary artery disease (CAD), or ischemic stroke causally increases circulating IL-6 or IL-6R. All instruments passed this directionality test (*P* < 0.05 for all comparisons).

#### Outcome data sources

All-cause mortality data were derived from the FinnGen study, which includes 356,077 Finnish individuals followed through national health registries [12].

A total of 57,224 deaths were recorded over a median follow-up of 11.7 years, providing high statistical power for causal inference.

### Causal effect on disease

#### Cardiovascular disease outcomes

Cardiovascular disease outcomes were obtained from large-scale genome-wide association studies and meta-analyses. Atrial fibrillation data were derived from the AFGen consortium including over 1 million individuals [13]. Coronary artery disease (CAD) data were obtained from the CARDIoGRAMplusC4D consortium meta-analysis including 185,000 CAD cases [14]. Stroke data were derived from the MEGASTROKE consortium including 521,612 individuals [15]. These datasets provided comprehensive coverage of major cardiovascular endpoints with substantial statistical power for detecting causal effects.

Similarly, other non-cardiovascular outcomes were tested. Among them there was a significant protective effect on cancer.

### Statistical analysis

#### Primary analysis methods

We performed inverse-variance weighted (IVW) Mendelian randomization as our primary analytical method, which provides the most precise estimates when all genetic instruments are valid [16]. The IVW method combines individual Wald ratio estimates using inverse-variance weighting.

Effect estimates were expressed as odds ratios (OR) for clinical outcomes, with 95% confidence intervals (CI) calculated using the delta method.

#### Power calculations

Statistical power was calculated using the mRnd online calculator (https://shiny.cnsgenomics.com/mRnd/) based on the proportion of variance explained by genetic instruments (R^2^), sample sizes, and expected effect sizes. For IL6R, with R^2^ = 4.2% and outcome sample sizes ranging from 400,000 to over 1 million individuals for cardiovascular outcomes, we had >99% power to detect odds ratios of 0.95–0.97 for cardiovascular disease outcomes and mortality. For multi-variant instruments, power calculations incorporated the combined R^2^ values.

#### Sensitivity analyses and pleiotropy assessment

To assess the robustness of our findings and potential violations of MR assumptions, we conducted multiple sensitivity analyses using different methodological approaches.

MR-Egger Regression: This method provides estimates robust to horizontal pleiotropy by allowing for a non-zero intercept. A significant intercept (*P* < 0.05) indicates directional pleiotropy. MR-Egger estimates were calculated using the mr_egger_regression function with bootstrap confidence intervals (*n* = 1,000 iterations).

MR-PRESSO (Pleiotropy RESidual Sum and Outlier): This method identifies and corrects for horizontal pleiotropy by detecting outlying genetic variants [17]. The analysis includes three components: (1) global test for horizontal pleiotropy, (2) outlier detection and removal, and (3) distortion test comparing estimates before and after outlier removal. We used the mr_presso function with 5,000 iterations and significance threshold of *P* < 0.05.

Heterogeneity Assessment: We assessed heterogeneity using Cochran’s Q statistic. Cochran’s Q follows a chi-squared distribution with degrees of freedom equal to the number of instruments minus one.

Leave-One-Out Analysis: We systematically excluded each genetic instrument and recalculated MR estimates to identify variants driving the overall association. This analysis used the mr_leaveoneout function, with results visualized using forest plots.

Expression Quantitative Trait Loci (eQTL) Analysis: We investigated tissue-specific expression effects using data from the Genotype-Tissue Expression (GTEx) project version 8, which includes 54 tissue types from 838 individuals [18]. eQTL data were accessed through the GTEx Portal API and analyzed using the gtexr package. We focused on cis-eQTLs (within 1 Mb of the transcription start site) with false discovery rate (FDR) <0.05.

All statistical analyses were performed using R version 4.3.2 (R Foundation for Statistical Computing, Vienna, Austria). The primary MR analyses utilized the TwoSampleMR package and MendelianRandomization package. Linkage disequilibrium pruning was conducted using PLINK v1.90 (https://www.cog-genomics.org/plink/) with the 1000 Genomes Project Phase 3 European reference panel.

### Data availability statement

Summary statistics used in this study are publicly available from the respective GWAS consortia and can be accessed through their designated repositories. GTEx data are available through the GTEx Portal (gtexportal.org) and dbGaP. Analysis code and supplementary data files are available upon reasonable request to the corresponding author.

## RESULTS

### Characteristics and validation of genetic instruments

All genetic instruments demonstrated adequate strength for Mendelian randomization analysis, with F-statistics well above the threshold of 10. Main individual SNPs characteristics are reported in Supplementary Table 1. The IL6R instrument set comprised nine independent genetic variants with a mean combined F-statistic of 343.61. The multi-variant instruments for other biomarkers achieved combined F-statistics well above the threshold of 10.

### Primary mendelian randomization results

#### IL6R protective effects

Genetically proxied higher IL6R levels were significantly associated with a lower risk of all-cause mortality (OR = 0.95; 95% CI: 0.91–0.98; *P* = 0.007; Figure 1), as well as with a reduced risk of major cardiovascular outcomes. These findings were derived from a Mendelian randomization analysis using genome-wide significant variants robustly associated with circulating IL6R levels.

**Figure 1:**
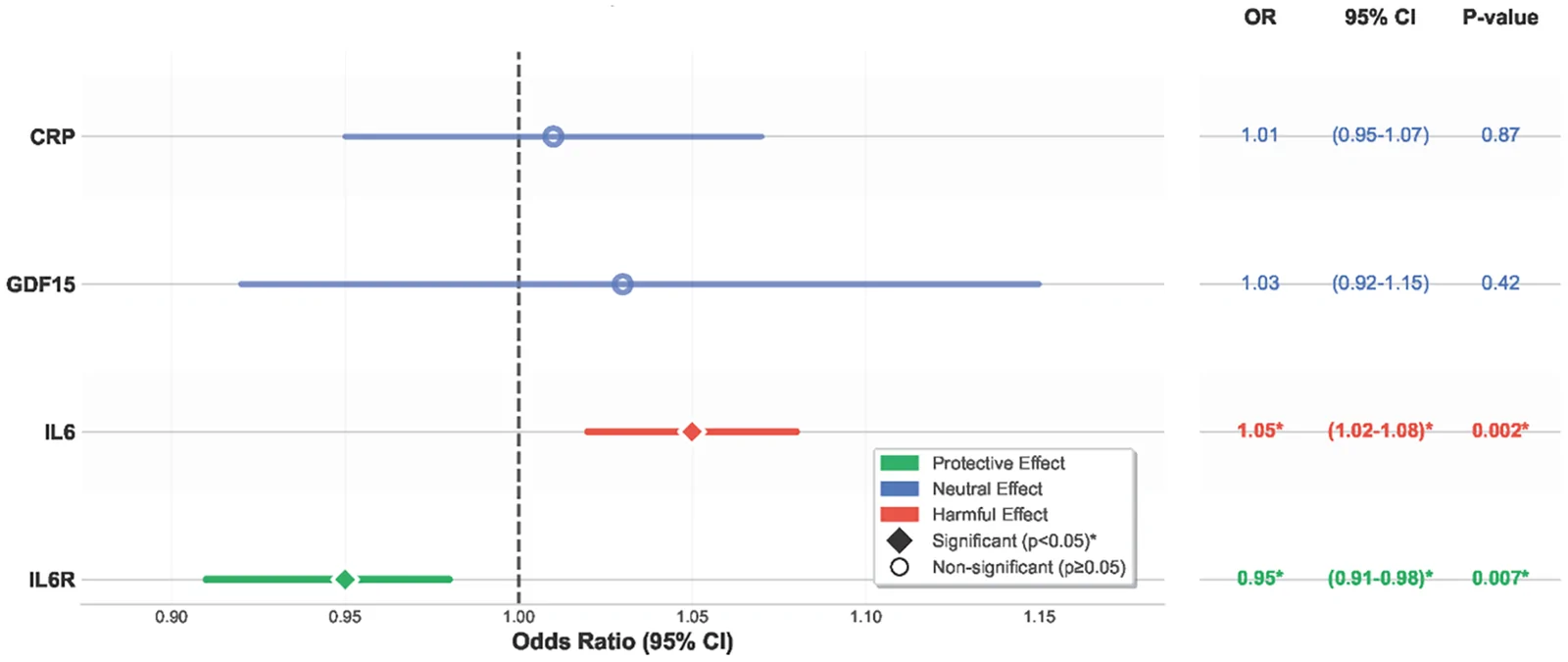
**Causal effects of inflammatory biomarkers on all-cause mortality.** Forest plot showing that higher IL6R is protective against mortality, whereas higher IL6 is harmful. CRP and GDF-15 are neutral. Abbreviations: IL6R: interleukin-6 receptor; IL6: interleukin-6; CRP: C-reactive protein; GDF-15: growth differentiation factor-15; OR: odds ratio; CI: confidence interval.

To further validate these results and to isolate potential *cis*-acting biological effects, we conducted a *cis*-Mendelian randomization analysis restricted to two independent variants located within the IL6R gene locus. This focused analysis confirmed the protective association with mortality (Supplementary Figure 1), reinforcing the causal relevance of IL6R in human survival and cardiovascular health.

Consistent protective effects were also observed for specific cardiovascular outcomes, including atrial fibrillation (OR = 0.96; 95% CI: 0.95–0.97; *P* < 0.001), coronary artery disease (OR = 0.97; 95% CI: 0.95–0.98; *P* < 0.001), and stroke (OR = 0.98; 95% CI: 0.97–1.0; *P* = 0.045) (Figure 2).

**Figure 2:**
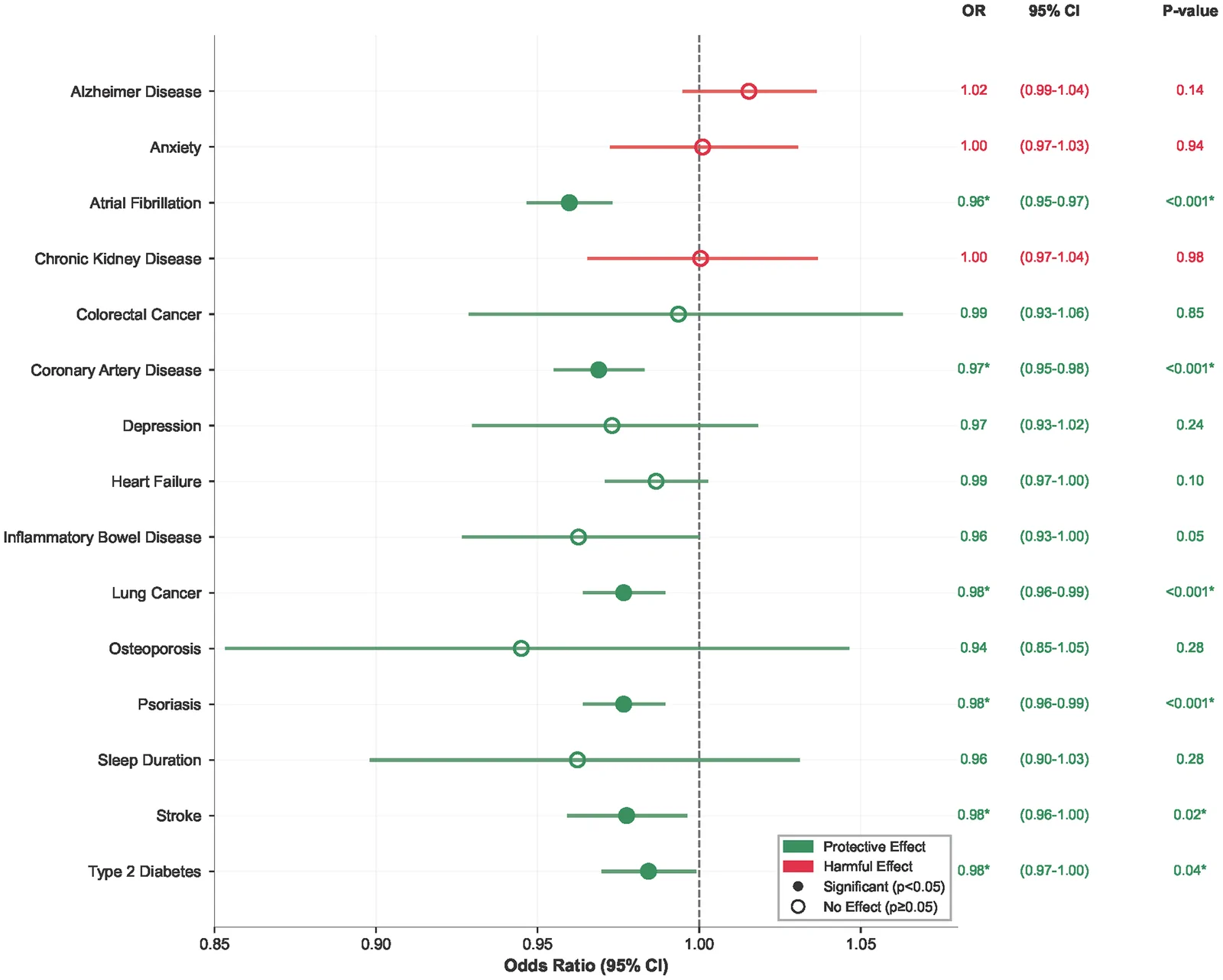
**IL6R effects on cardiovascular disease outcomes.** Forest plot showing protective effects of genetically predicted IL6R levels on major cardiovascular and non-cardiovascular outcomes. Abbreviations: OR: odds ratio; CI: confidence interval.

#### IL6 harmful effects

Genetically predicted higher IL6 levels showed significant harmful effects on mortality (OR 1.05, 95% CI 1.02-1.08, *P* = 0.002) (Figure 1). Sensitivity analyses confirmed these findings with no evidence of pleiotropy.

Reverse MR provided no evidence that genetic liability to CAD, AF, or ischemic stroke causally affects IL6 or IL6R: CAD and IL6 OR 1.03 (95% CI 0.95–1.12), *P* = 0.48; AF and IL6 OR 1.49 (95% CI 0.00–1097.74), *P* = 0.91; stroke and IL6 OR 1.03 (95% CI 0.97–1.10), *P* = 0.35; CAD and IL-6R OR 0.80 (95% CI 0.62–1.03), *P* = 0.10; AF and IL6R OR 0.49 (95% CI 0.00–66.81), *P* = 0.78; stroke and IL6R OR 0.96 (95% CI 0.89–1.04), *P* = 0.32. Sensitivity analyses were concordant and did not indicate directional pleiotropy.

#### CRP and GDF15

Neither CRP nor GDF15 demonstrated significant causal effects on mortality or cardiovascular disease outcomes. CRP showed neutral associations (mortality OR 1.01, *P* = 0.87, Figure 2), as did GDF15 (mortality OR 1.03, *P* = 0.42).

Individual SNP analysis confirmed the robustness of our findings across all nine IL6R genetic instruments (Figure 3). The consistent directional effects observed in both IL6 and IL6R analyses, with opposite biological directions, further validate the causal relationships identified in our primary analyses.

**Figure 3:**
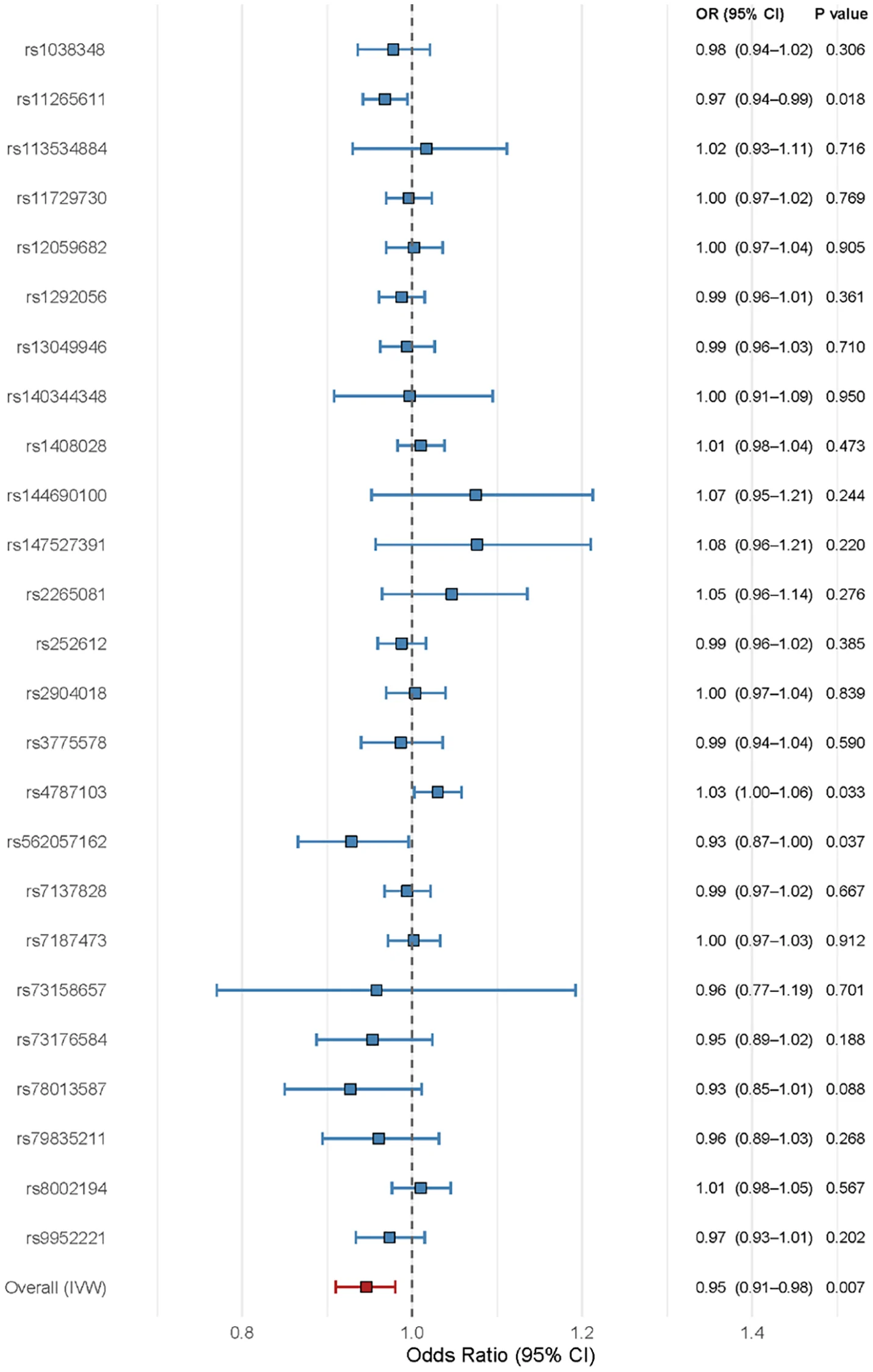
**Individual IL6R SNP analysis.** Forest plot of genome-wide significant IL6R variants on all-cause mortality showing individual SNP effects. Abbreviation: SNP: single nucleotide polymorphism; remaining abbreviations as Figure 1.

#### Expression quantitative trait loci analysis

eQTL analysis confirmed the biological relevance of our genetic instruments through their effects on target gene expression (Figure 4). Selected IL6R variants showed significant negative eQTL effects, indicating reduced IL6R gene expression, in line with their protective association with mortality.

**Figure 4:**
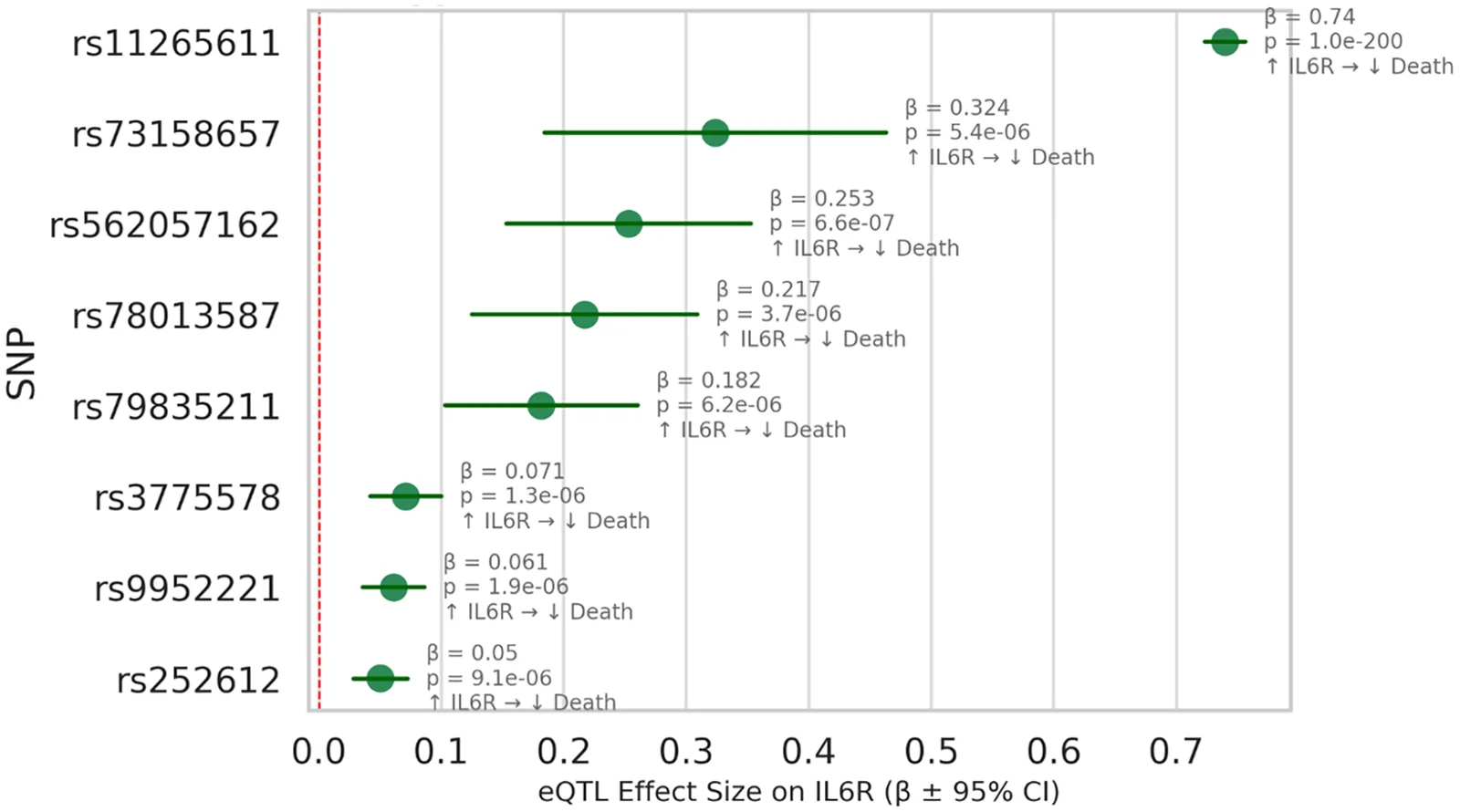
**IL6R eQTL effects supporting protective association with mortality.** The data are derived from GTEx v8 (Whole Blood) and large-scale GWAS consortia. This forest plot displays the cis-eQTL effects of genetic variants used as instrumental variables in our Mendelian randomization analysis of IL6R expression and all-cause mortality. Each row corresponds to a single nucleotide polymorphism (SNP), with effect sizes (β) representing the change in IL6R gene expression per allele, and horizontal bars indicating 95% confidence intervals. The red vertical line indicates no effect (β = 0). These results provide functional support for a protective role of higher IL6R expression in reducing mortality, and confirm the biological validity of the selected instruments. Abbreviations: eQTL: expression quantitative trait loci; SNP: single nucleotide polymorphism; remaining abbreviations as Figure 1.

#### Sensitivity analyses

Sensitivity analyses supported the robustness of these findings: Cochran’s *Q*-test showed no significant heterogeneity (IVW Q = 29.95, df = 25, *P* = 0.23), the MR-Egger intercept was close to zero and non-significant (−0.0034, SE = 0.0051, *P* = 0.51), leave-one-out analysis indicated that no single SNP drove the association (Supplementary Figure 2), and the funnel plot showed no evidence of asymmetry (Supplementary Figure 3). In addition, the scatter plot of SNP-specific effects demonstrated consistent alignment of variant estimates along the slope of the causal effect (Supplementary Figure 4), providing visual confirmation of the direction and magnitude of the protective association. Collectively, these results confirm a consistent and unbiased relationship between higher IL6R levels and reduced mortality risk.

## DISCUSSION

This large-scale Mendelian randomization study provides robust genetic evidence that inflammatory biomarkers causally influence long-term human survival (Figure 5).

**Figure 5:**
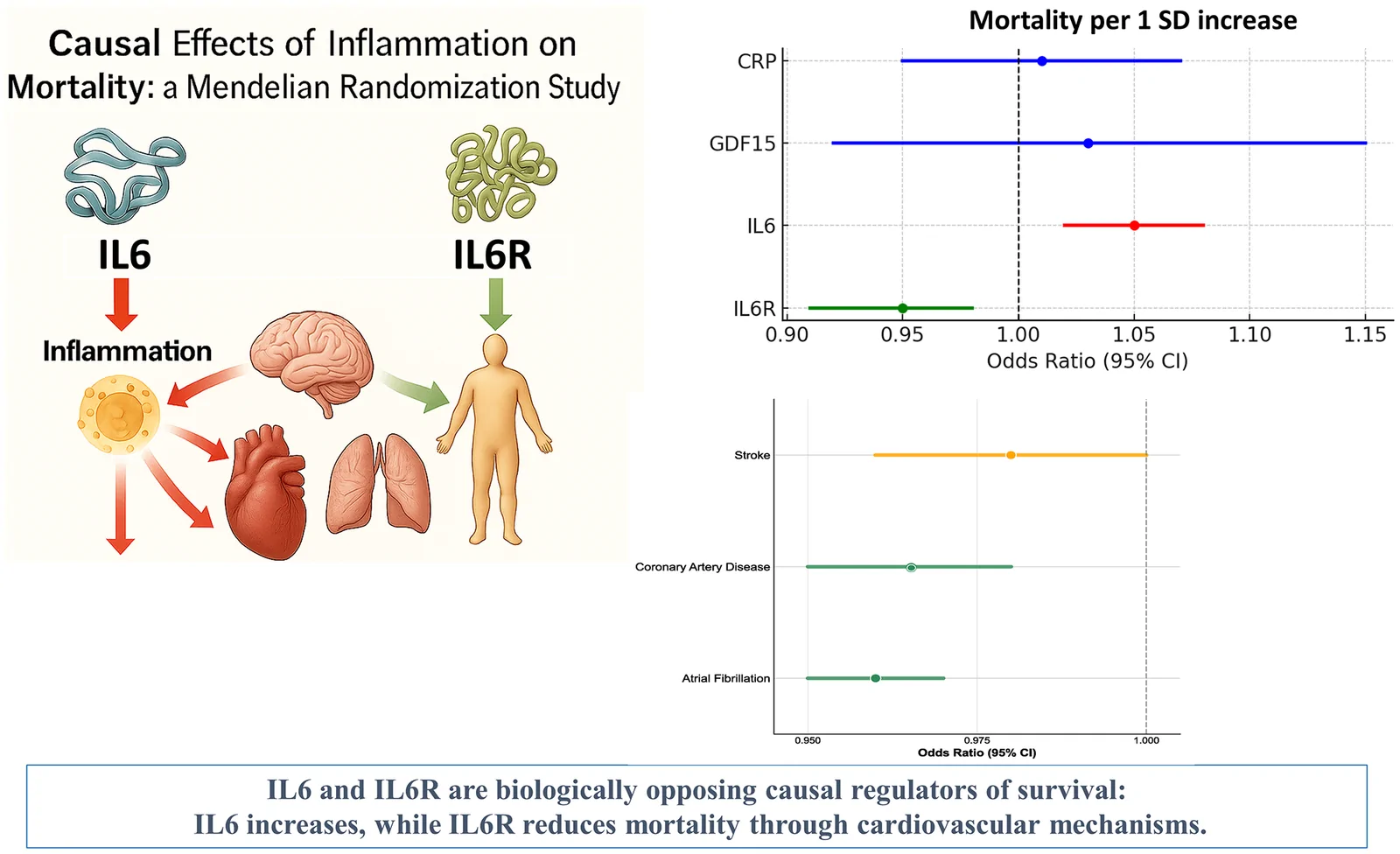
This illustration summarizes the Mendelian randomization findings on the causal impact of inflammatory biomarkers—IL6, IL6R, CRP, and GDF15—on all-cause mortality in over 750,000 genotyped individuals.

To our knowledge, this is the first MR analysis specifically focusing on the IL6/IL6R signaling pathway with long-term mortality as the primary outcome.

Using data from more than 750,000 individuals, we demonstrate that the two central components of this axis exert opposite effects: genetically higher soluble IL6 receptor (sIL6R) levels are protective, whereas elevated IL6 levels increase mortality risk [19].

A genetically predicted one–standard-deviation increase in sIL6R was associated with a ~5% reduction in all-cause mortality, while the same increment in IL6 corresponded to an increased mortality risk. The protective effect of sIL6R appears partly mediated by its favorable impact on major cardiovascular outcomes, including atrial fibrillation, coronary artery disease, and stroke. These findings are consistent with IL6R’s established role in modulating vascular inflammation and atherosclerosis. Beyond cardiovascular endpoints, we also observed a significant reduction in lung cancer, suggesting broader anti-inflammatory benefits.

The distinct directions of effect likely reflect different biological mechanisms. IL6R variants capture physiological modulation of receptor shedding, which may reduce IL6-mediated inflammation, whereas IL6 variants mirror sustained pro-inflammatory states that promote vascular injury and thrombosis [15, 17]. This targeted modulation of the IL6 pathway may offer a safer and more effective strategy for prevention than broad anti-inflammatory approaches. The cardiovascular and cancer protective effects of IL6R may offer a mechanistic explanation for its mortality benefits: by reducing atrial fibrillation, coronary artery disease, and stroke risk, IL6R directly addresses the leading causes of cardiovascular mortality. This cardiovascular-mediated protection offers a more targeted, potentially safer therapeutic avenue than broad anti-inflammatory approaches. In contrast, we found no evidence for causal effects of CRP or GDF-15 on mortality or cardiovascular disease, despite their strong epidemiological associations with cardiovascular outcomes [10, 20]. These findings underscore the importance of distinguishing prognostic correlates from true biological effectors, and suggests that these biomarkers may act as downstream indicators rather than upstream mediators of cardiovascular pathology.

Taken together, the results of the current mendelian randomization study strengthen the rationale for IL6R antagonism as a potential strategy to reduce cardiovascular disease and associated mortality.

The protective effects of sIL6R observed here are biologically plausible and mechanistically coherent with cardiovascular pathophysiology [19]. IL6 is a key driver of inflammation [4, 21]. The IL6R variants used as instruments increase the amount of receptor detectable in blood while proportionally reducing the receptor present on cell surfaces [22]. In vascular and myocardial tissues—where surface IL6R is naturally scarce—this shift, together with plasma regulators that bind IL-6–receptor complexes, dampens downstream IL6 activity and lowers inflammatory burden at the vessel wall and myocardium. In cardiovascular tissues, elevated IL-6R appears to exert anti-inflammatory effects by sequestering IL-6 and preventing its engagement with membrane-bound receptors, thereby reducing vascular inflammation, endothelial dysfunction, and thrombotic risk. The cardiovascular-specific protective effects we observed—particularly for atrial fibrillation and coronary artery disease—align with IL6R’s established role in modulating cardiac electrophysiology and coronary atherosclerosis. These cardiovascular benefits provide a clear mechanistic pathway through which IL6R reduces overall mortality, as cardiovascular disease remains the leading cause of death globally [23, 24].

Conversely, the harmful effects of IL6 are consistent with its established role as a pro-inflammatory cytokine with direct cardiovascular toxicity. IL6 promotes acute-phase responses, activates multiple inflammatory cascades, and contributes to insulin resistance, endothelial dysfunction, and thrombotic processes that directly contribute to cardiovascular disease development [6, 25]. Elevated IL6 has been linked to increased risk of myocardial infarction, stroke, and heart failure through its effects on coronary plaque instability, cardiac remodeling, and arrhythmogenesis, supporting its role as a causal mediator of cardiovascular morbidity and mortality [26].

Sensitivity analyses across multiple MR approaches yielded consistent findings for IL6R, with significant protective effects in all tested methods and minimal heterogeneity, reinforcing the robustness of the results. These genetic data align with clinical trial evidence: IL6R antagonists such as tocilizumab have demonstrated survival benefits in several inflammatory conditions, including COVID-19, without major long-term safety concerns in approved indications [27, 28].

Our findings suggest that precision targeting of IL6 signaling—particularly through selective modulation of trans-signaling—may represent a promising and safe strategy for preventing cardiovascular disease and reducing mortality risk. The cardiovascular-specific benefits we identified provide a strong therapeutic rationale for IL6R-targeted interventions in high-risk populations. However, translating these findings into preventive clinical applications will require careful risk–benefit evaluation and rigorous long-term safety assessments in dedicated cardiovascular prevention trials.

### Limitations

Our study has limitations. First, analyses were restricted to individuals of European ancestry, which may limit generalizability to other populations with different genetic backgrounds. Second, while we assessed major cardiovascular outcomes, we did not examine other potential mediating pathways such as metabolic or inflammatory diseases that could contribute to IL6R’s mortality effects.

## CONCLUSIONS

This Mendelian randomization study demonstrates that the IL6/IL6R axis has a causal impact on human survival through cardiovascular mechanisms: IL6R exerts a protective effect, whereas IL6 is detrimental. The protective influence of IL6R on atrial fibrillation, coronary artery disease, stroke and lung cancer provides a mechanistic basis for its mortality benefits. Concordant genetic and pharmacological evidence positions IL6R as a promising therapeutic target for cardiovascular disease prevention, while the neutral findings for CRP and GDF15 support their role as biomarkers rather than causal drivers. Collectively, these results identify the IL6 pathway as a key translational target for reducing cardiovascular disease and mortality.

## Supplementary Materials

Supplementary Figures

Supplementary Table 1
