# Supplementary material for: Causal effects of inflammation on long-term mortality: A mendelian randomization study
Source: Aging (Albany NY). 2026 Feb 5;18(1):44–58. doi: 10.18632/aging.206352 (PMC13632775; doi:10.18632/aging.206352)
Supplement: Supplementary file 1 [file aging-18-1-206352-s001.pdf]

SUPPLEMENTARY FIGURES

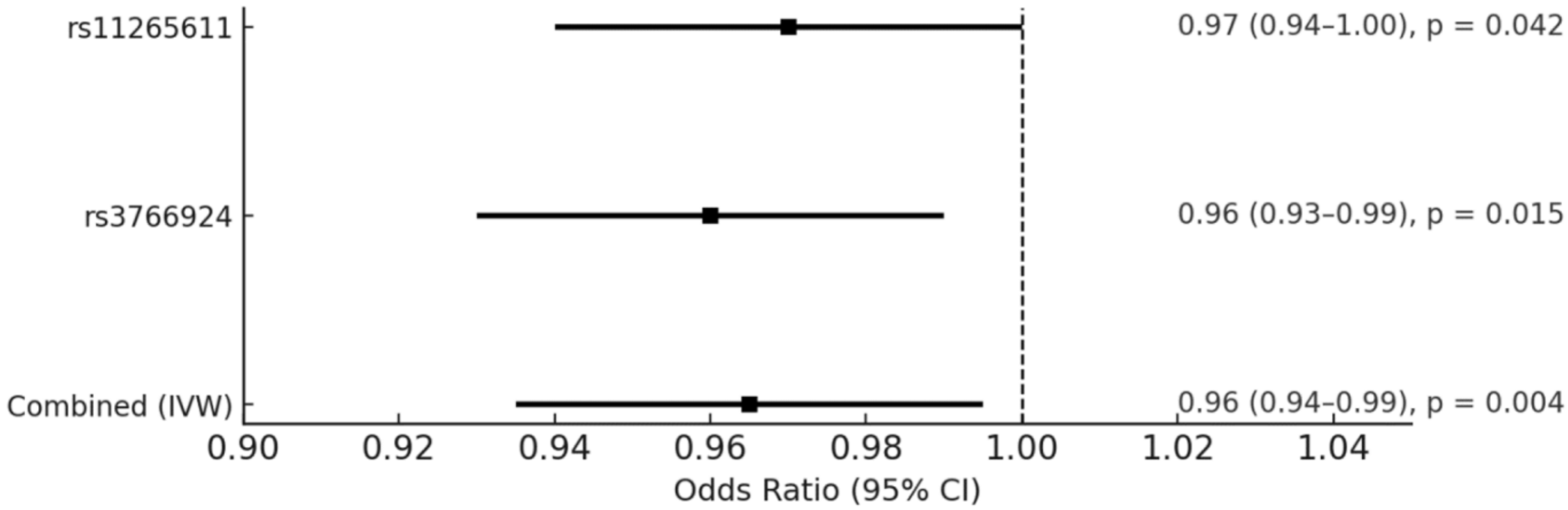

Supplementary Figure 1. Cis-mendelian randomization for IL6R and mortality.

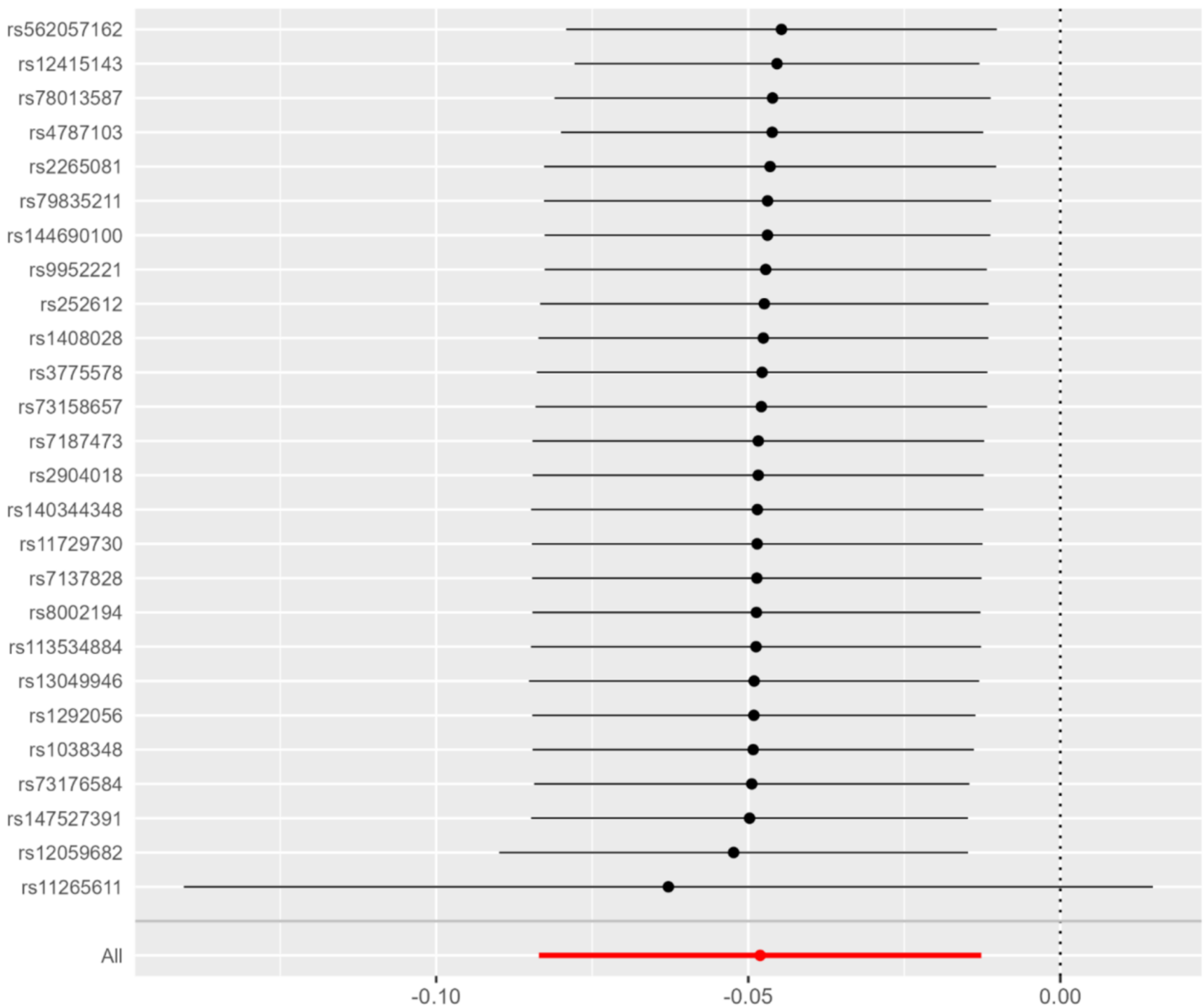

Supplementary Figure 2. Leave-one-out analysis.

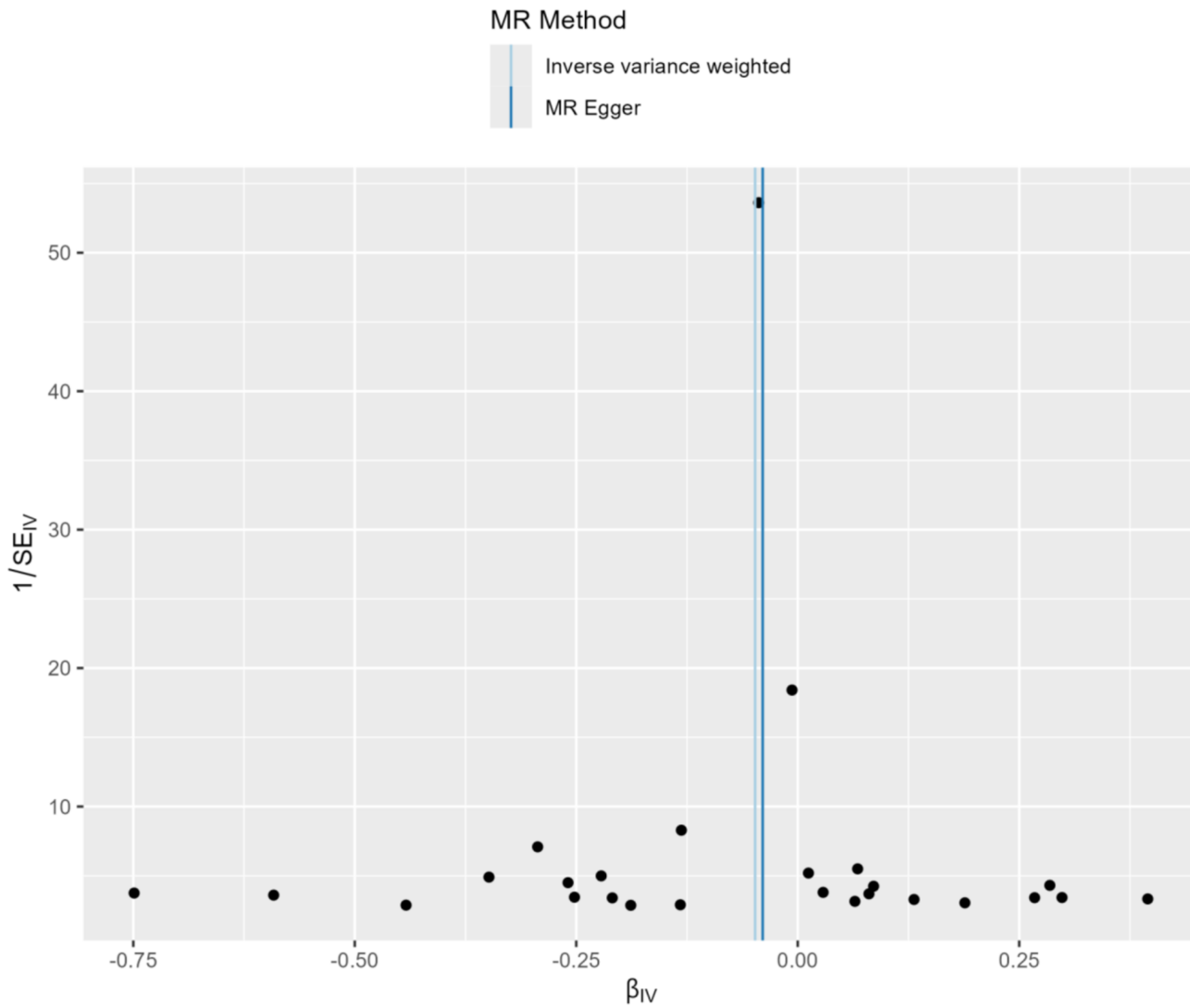

Supplementary Figure 3. Funnel plot.

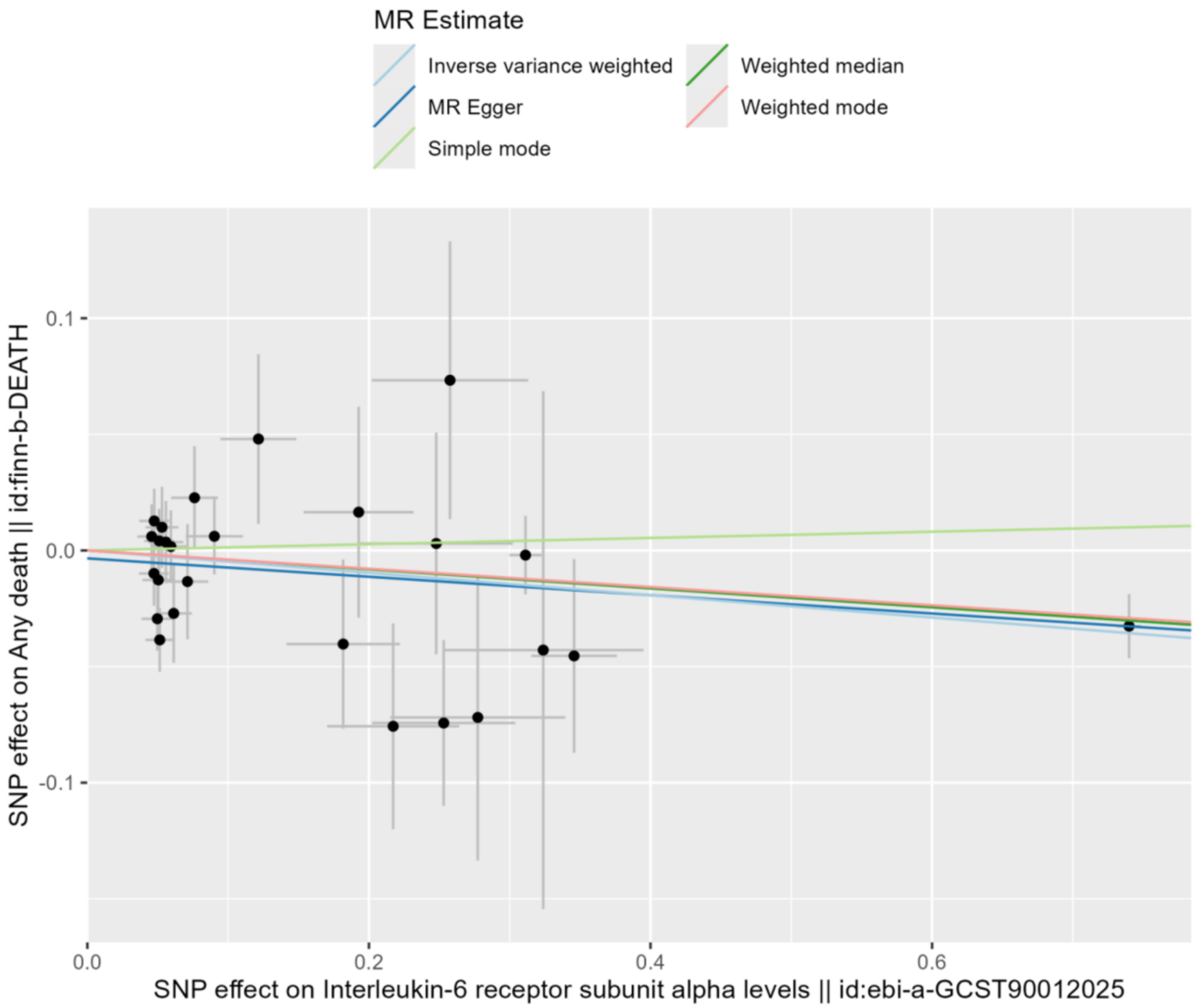

Supplementary Figure 4. Scatter plot.
