# Supplementary material for: Causal effects of inflammation on long-term mortality: A mendelian randomization study
Source: Aging (Albany NY). 2026 Feb 5;18(1):44–58. doi: 10.18632/aging.206352 (PMC13632775; doi:10.18632/aging.206352)
Supplement: Supplementary file 2 [file aging-18-1-206352-s002.pdf]

SUPPLEMENTARY TABLE

Supplementary Table 1. Individual SNPs characteristics for IL-6 receptor.

SNP	Effect allele	Other allele	EAF	Beta	SE	F-statistic
**rs1038348**	A	G	0.0824	−0.0761	0.0166	21.016149
**rs11265611**	A	G	0.5865	0.7399	0.0085	7577.19045
**rs113534884**	A	G	0.0318	0.1927	0.0391	24.289016
**rs11729730**	A	G	0.4711	−0.051	0.0106	23.148807
**rs12059682**	C	T	0.218	−0.3112	0.0115	732.290662
**rs1292056**	T	C	0.4376	−0.0475	0.0106	20.080545
**rs13049946**	C	T	0.5432	−0.0902	0.0203	19.743357
**rs140344348**	C	T	0.0143	−0.2478	0.0544	20.749365
**rs1408028**	G	A	0.4532	−0.0473	0.0106	19.911801
**rs144690100**	A	G	0.014	−0.2773	0.0622	19.875542
**rs147527391**	A	G	0.0165	0.2576	0.0555	21.542979
**rs2265081**	A	C	0.9629	−0.3457	0.0304	129.3158
**rs252612**	C	T	0.3126	0.0504	0.0113	19.893179
**rs2904018**	A	G	0.8241	0.0558	0.0123	20.580607
**rs3775578**	C	T	0.1418	0.0711	0.0147	23.394002
**rs4787103**	G	A	0.4008	−0.0497	0.0112	19.691406
**rs562057162**	G	A	0.0267	0.2531	0.0509	24.725707
**rs7137828**	T	C	0.5378	−0.0457	0.0102	20.073914
**rs7187473**	C	T	0.262	0.0593	0.0121	24.0181
**rs73158657**	T	C	0.0128	0.3238	0.0712	20.682056
**rs73176584**	C	T	0.0497	−0.1215	0.0269	20.400837
**rs78013587**	T	C	0.0161	0.2172	0.0469	21.447366
**rs79835211**	G	A	0.0236	0.1817	0.0402	20.429501
**rs8002194**	C	T	0.2451	0.053	0.0116	20.875446
**rs9952221**	A	T	0.1495	0.0613	0.0129	22.580915
